# Cytomegalovirus-associated splenic infarcts in a female patient with Factor V Leiden mutation: a case report

**DOI:** 10.1186/1752-1947-2-385

**Published:** 2008-12-16

**Authors:** Lihi Atzmony, Nili Saar, Tamar Chundadze, Yaron Arbel, Dan Justo, Noa Mashav

**Affiliations:** 1 Department of Internal Medicine D, Tel-Aviv Sourasky Medical Center, 6 Weitzman Street, Tel-Aviv 64239, Israel

## Abstract

**Introduction:**

Cytomegalovirus-associated thrombosis has rarely been reported in the medical literature, and if so, mainly in immunocompromized patients.

**Case presentation:**

We report the case of a 36-year-old Caucasian woman with acute cytomegalovirus infection presenting with spontaneous splenic infarcts. Trans-esophageal echocardiography did not show any vegetations or mural thrombi. The patient was also found to be heterozygous for the Factor V Leiden mutation. Anticoagulation treatment was considered but ruled out since cytomegalovirus was the obvious trigger for thrombosis in this patient. To the best of our knowledge, this is only the third report to date of cytomegalovirus-associated splenic infarcts.

**Conclusion:**

This case report serves as additional evidence for the role of cytomegalovirus in thrombosis.

## Introduction

Cytomegalovirus (CMV)-associated thrombosis has only rarely been reported in the medical literature. While most reports of CMV-associated thrombosis discuss immunocompromized transplant recipients or HIV-positive patients [1], we report a case of acute CMV infection in an immunocompetent patient, presenting with spontaneous spleen infarcts. This case report serves as additional evidence for the role of CMV in thrombosis.

## Case presentation

A previously healthy, 36-year-old Caucasian woman, presented with a 2-week history of high-grade fever, epigastric pain, diarrhea, headache and 6-kg weight loss. During this period, she had daily documented temperatures of 37°C to 39.5°C. At the point of admission, the patient was not receiving any prescription drugs or taking oral contraceptives regularly. She worked at a pet shop, was not a regular user of tobacco products, drugs or alcohol, had no recent contact with people who were ill and she had not traveled in the preceding months.

Physical examination showed an oral temperature of 37°C and a pulse rate of 106 beats per minute. Cardiac auscultation revealed no murmurs. Lung auscultation was unremarkable and hepato-splenomegaly was palpable on abdominal examination. There was no lymphadenopathy or meningismus on physical examination. A papular rash was apparent on her lower limbs.

**Figure 1:**
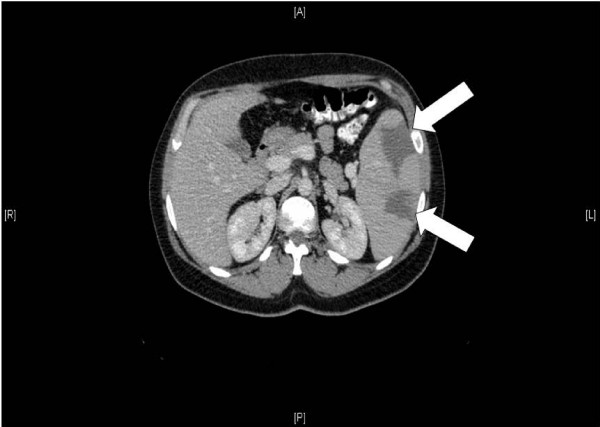
**Two large splenic infarcts, as demonstrated on an abdominal CT scan (white arrows)**.

Laboratory test results demonstrated a white blood cell count of 4,500 per mm^3 ^with 42% lymphocytes. Atypical lymphocytes were observed on a blood smear. Alanine transaminase and aspartate transaminase levels were mildly elevated (59 and 64 U per Litre, respectively). An abdominal CT scan demonstrated hepato-splenomegaly with multiple splenic infarcts (Figure 1). No splenic artery or vein thromboses were demonstrated. Blood and stool cultures were sterile and trans-esophageal echocardiography did not show any vegetations or mural thrombi.

Serologic tests for the Epstein-Barr virus (EBV) were consistent with IgG antibodies; serologic tests for Hepatitis B, Hepatitis C and HIV were negative, while serologic tests for CMV IgM antibodies were positive on two occasions. A positive CMV PP65 antigenemia assay confirmed the diagnosis of acute CMV infection, but a prior hypercoagulable state was still considered. PT and aPTT values were within normal limits. Lupus-anticoagulant and anti-cardiolipin antibodies were not detected. Anti-thrombin III, protein C and protein S levels were within normal range. The prothrombin G20210A mutation was excluded, while the patient was found to be heterozygous for the Factor V Leiden mutation. Serologic tests for CMV revealed positive IgG antibodies, consistent with seroconversion.

At this point, anticoagulation treatment was considered, but it was ruled out since CMV was the obvious trigger or cause for thrombosis in this patient. Ganciclovir treatment was also considered because of her grave condition, but it was ruled out because over the next few days her fever declined and the papular rash resolved spontaneously.

An abdominal ultrasound revealed no significant changes in spleen size and infarct presence a few days later, and she was subsequently discharged from hospital without any treatment. Two months later, she became asymptomatic.

## Discussion

Unexplained fever accompanied by splenic infarcts in an immunocompetent patient can be seen in endocarditis, in viral infections such as EBV, in infectious vasculitis as observed in neisserial infections, and in various other non-infectious conditions, including sickle-cell anemia, autoimmune vasculitis and hypercoagulable states [2].

Here, we report the case of an immunocompetent female patient with concomitant acute CMV infection and thrombosis, a syndrome reported previously in medical literature mainly in immunocompromized patients. Only very few reports on concomitant acute CMV infection and thrombosis in immunocompetent patients exist [1]. Moreover, reports on acute CMV infection and splenic infarcts in immunocompetent patients are even more rare [3,4]. To the best of our knowledge, this is only the third report ever of CMV-associated splenic infarcts in an immunocompetent patient.

The exact pathologic mechanism by which CMV triggers thrombosis is still unclear. Current theories suggest that CMV induces thrombosis by enhancing platelet and leukocyte adhesion to infected endothelial cells, or, alternatively, by increasing the circulatory levels of Factor VIII. Other theories suggest that CMV induces transient antiphospholipid antibody production and enhances vascular smooth-muscle proliferation [1]. Genetic predisposing factors for thrombosis in patients with CMV-associated thrombosis, such as Factor V Leiden mutation, were also previously reported [5]. It is still difficult, however, to determine whether CMV was the direct cause of thrombosis in the patient presented here, or whether CMV simply served as a precipitating factor for thrombosis in a patient with thrombogenic tendency.

## Conclusion

CMV is a rare, but potentially significant, cause or precipitating factor for thrombosis in immunocompetent hosts. We think that all patients with unexplained fever and spontaneous thrombosis should be screened for CMV infection; in cases of splenic infarcts due to thromboembolism or congenital hypercoagulability, anticoagulation is mandatory, while in cases of splenic infarcts alone, CMV infection may influence the decision on whether or not to start anticoagulation therapy.

## Abbreviations

CMV: Cytomegalovirus; EBV: Epstein-Barr virus

## Consent

Written informed consent was obtained from the patient for publication of this case report and accompanying images. A copy of the written consent is available for review by the Editor-in-Chief of this journal.

## Competing interests

The authors declare that they have no competing interests.

## Authors' contributions

LA, DJ and NM analyzed and interpreted the patient data. NS, TC and YA wrote the manuscript. All authors read and approved the final manuscript.
